# A graph-theoretical approach in brain functional networks. Possible implications in EEG studies

**DOI:** 10.1186/1753-4631-4-S1-S8

**Published:** 2010-06-03

**Authors:** Fabrizio De Vico Fallani, Luciano da Fontoura Costa, Francisco Aparecido Rodriguez, Laura Astolfi, Giovanni Vecchiato, Jlenia Toppi, Gianluca Borghini, Febo Cincotti, Donatella Mattia, Serenella Salinari, Roberto Isabella, Fabio Babiloni

**Affiliations:** 1 IRCCS "Fondazione Santa Lucia", Rome, Italy; 2 Department of Human Physiology and Pharmacology, University “Sapienza”, Rome, Italy; 3 Department of Informatica e Sistemistica, University “Sapienza”, Rome, Italy; 4 “2° Div. (Relazioni Internazionali) della Direzione Generale della Sanità Militare”, Rome, Italy; 5 Institute of Physics at Sao Carlos, University of Sao Paulo, Sao Carlos-SP, Brazil

## Abstract

**Background:**

Recently, it was realized that the functional connectivity networks estimated from actual brain-imaging technologies (MEG, fMRI and EEG) can be analyzed by means of the graph theory, that is a mathematical representation of a network, which is essentially reduced to nodes and connections between them.

**Methods:**

We used high-resolution EEG technology to enhance the poor spatial information of the EEG activity on the scalp and it gives a measure of the electrical activity on the cortical surface. Afterwards, we used the Directed Transfer Function (DTF) that is a multivariate spectral measure for the estimation of the directional influences between any given pair of channels in a multivariate dataset. Finally, a graph theoretical approach was used to model the brain networks as graphs. These methods were used to analyze the structure of cortical connectivity during the attempt to move a paralyzed limb in a group (N=5) of spinal cord injured patients and during the movement execution in a group (N=5) of healthy subjects.

**Results:**

Analysis performed on the cortical networks estimated from the group of normal and SCI patients revealed that both groups present few nodes with a high out-degree value (i.e. outgoing links). This property is valid in the networks estimated for all the frequency bands investigated. In particular, cingulate motor areas (CMAs) ROIs act as ‘‘hubs’’ for the outﬂow of information in both groups, SCI and healthy. Results also suggest that spinal cord injuries affect the functional architecture of the cortical network sub-serving the volition of motor acts mainly in its local feature property.

In particular, a higher local efficiency *E_l_* can be observed in the SCI patients for three frequency bands, theta (3-6 Hz), alpha (7-12 Hz) and beta (13-29 Hz).

By taking into account all the possible pathways between different ROI couples, we were able to separate clearly the network properties of the SCI group from the CTRL group. In particular, we report a sort of compensatory mechanism in the SCI patients for the Theta (3-6 Hz) frequency band, indicating a higher level of “activation” *Ω* within the cortical network during the motor task. The activation index is directly related to diffusion, a type of dynamics that underlies several biological systems including possible spreading of neuronal activation across several cortical regions.

**Conclusions:**

The present study aims at demonstrating the possible applications of graph theoretical approaches in the analyses of brain functional connectivity from EEG signals. In particular, the methodological aspects of the i) cortical activity from scalp EEG signals, ii) functional connectivity estimations iii) graph theoretical indexes are emphasized in the present paper to show their impact in a real application.

## Background

Over the last decade, there has been a growing interest in the detection of the functional connectivity in the brain from different neuroelectromagnetic and hemodynamic signals recorded by several neuro-imaging devices such as the functional Magnetic Resonance Imaging (fMRI) scanner, electroencephalography (EEG) and magnetoencephalography (MEG) apparatus. Many methods have been proposed and discussed in the literature with the aim of estimating the functional relationships among different cerebral structures [1-5]. However, the necessity of an objective comprehension of the network composed by the functional links of different brain regions is assuming an essential role in the Neuroscience. The extraction of salient characteristics from brain connectivity patterns is an open challenging topic, since often the estimated cerebral networks have a relative complex structure. Recently, it was realized that the functional connectivity networks estimated from actual brain-imaging technologies (MEG, fMRI and EEG) can be analyzed by means of the graph theory [6,7]. In those studies, the authors have evaluated two characteristic measures, the average shortest path L and the clustering coefficient C, to extract respectively the global and local properties of the network structure. They have found that anatomical brain networks exhibit a high degree to which nodes tend to cluster together (i.e. a high C) and a relatively short distance between all the nodes (i.e. a low L). These values identify a particular model that interpolate between a regular lattice and a random structure. Such a model has been designated as “small-world” network in analogy with the concept of the small-world phenomenon observed more than 30 years ago in social systems [8]. In a similar way, many types of functional brain networks have been analyzed according to this mathematical approach. In the functional brain connectivity context, these properties have been demonstrated to reflect an optimal architecture for the information processing and propagation among the involved cerebral structures. However, the performance of cognitive and motor tasks as well as the presence of neural diseases has been demonstrated to affect such a small-world topology, as revealed by the significant changes of L and C [9-11].

The small-world concept in a complex network is strictly related to the length of the shortest paths within the network, which is given by the smallest number of edges needed to go from a starting vertex *i* to a target node *j *[12]. However, shortest paths just represent one possible way in which two nodes in the network can communicate and other existing pathways should be generally taken into account to characterize the connectivity pattern. In particular, by neglecting the longer pathways important information is lost about the alternative trails that could connect any two nodes in a network. This information appears strictly related to the concepts of “redundancy” and “robustness”, critical resources for the survival of many biological systems as they provide reliable function despite the death of individual elements. Indeed, the presence of more than one path between two nodes in the graph tends to increase the interaction between them, while enhancing the resilience to damages. In particular, the human brain is supposed to exhibit a high level of alternative anatomical and functional pathways between adjacent regions and sites. This type of organization would allow the brain to reshape its physiologic mechanisms in order to compensate the critical consequences of possible diseases [13].

Recently, an interesting methodology – the superedges approach - has been proposed [14] in physics to obtain a detailed analysis of networks considering the concept of generalized connectivity. This approach allows characterizing the networks properties by taking into account all the possible paths between pairs of nodes.

In order to illustrate the potential of the graph theoretical approach in the brain functional network analysis, we report the results obtained with a set of high-resolution *EEG* signals from spinal cord injured patients and control subjects during the preparation of an intended motor act.

## Methods

### Cortical activity estimation

High-resolution EEG technology involves the use of a larger number of scalp electrodes (64-256). In addition, high-resolution EEG uses realistic MRI-constructed subject head models and spatial de-convolution estimations, which are commonly computed by solving a linear inverse problem based on boundary-element mathematics [15,16]. In the present applications, the cortical activity was estimated from EEG recordings by using a realistic head model, whose cortical surface consisted of about 5000 triangles disposed uniformly.

Each triangle represents the electrical dipole of a particular neuronal population and the estimation of its current density was computed by solving the linear inverse problem (see following paragraphs). In this way, the electrical activity in different Regions Of Interest (ROIs) can be obtained by averaging the current density of the various dipoles within the considered cortical area.

### Head models and regions of interest

In order to estimate cortical activity from conventional EEG scalp recordings, realistic head models reconstructed from T1-weighted MRIs are employed. Scalp, skull and dura mater compartments are segmented from MRIs and tessellated with about 5000 triangles. Then, the cortical regions of interest (ROIs) are drawn by a neuroradiologist on the computer-based cortical reconstruction of the individual head model by following a Brodmann’s mapping criterion.

### Estimation of cortical source current density

The solution of the following linear system:

*Ax* = *b* + *n* (1)

provides an estimation of the dipole source configuration *x* which generates the measured EEG potential distribution *b*. The system includes also the measurement noise *n*, assumed to be normally distributed. *A* is the lead field matrix, where each *j-th* column describes the potential distribution generated on the scalp electrodes by the *j-th* unitary dipole. The current density solution vector *ξ* of Eq. 1 was obtained as:

 (2)

where *M*, *N* are matrices associated to the metrics of data and source space, respectively; *λ* is a regularization parameter; || … ||_M_ represent the M-norm of the data space *b* and || … ||_N_ the N-norm of the solutions space *x*. The formula 2 represents a minimization problem also known as *linear inverse* problem.

**Figure 1:**
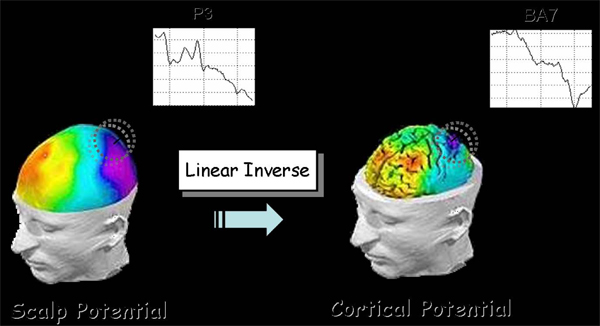
Electrical activity estimation in the Brodmann area 7 from the scalp measurement in the parietal sensor P3.

As a metric of the data space the identity matrix is generally employed. However, the metric in the source space can be opportunely modified when hemodynamic information is available from recorded fMRI data. This aspect can notably improve the localization of the source activity. An estimate of the signed magnitude of the dipolar moment for each one of the 5000 cortical dipoles was then obtained for each time point. The instantaneous average of all the dipoles’ magnitude within a particular ROI was used to deal with the average activity in that ROI during the whole time interval of the experimental task. Figure 1 illustrates the effect of the linear inverse problem’s solution. From a scalp potential distribution one can estimate accurately the original cortical potential.

### Functional connectivity estimation

Many EEG and/or MEG frequency-based methods that have been proposed in recent years for assessment of the directional influence of one signal on another are based mainly on the Granger theory of causality. Granger theory mathematically defines what a “causal” relation between two signals is. According to this theory, an observed time series x(n) is said to cause another series y(n) if the knowledge of x(n)’s past significantly improves prediction of y(n); this relation between time series is not necessarily reciprocal, i.e., x(n) may cause y(n) without y(n) causing x(n). This lack of reciprocity allows the evaluation of the direction of information flow between structures. Kaminski and Blinowska [3] proposed a multivariate spectral measure, called the Directed Transfer Function (DTF), which can be used to determine the directional influences between any given pair of channels in a multivariate dataset. DTF is an estimator that simultaneously characterizes the direction and spectral properties of the interaction between brain signals and requires only one multivariate autoregressive (MVAR) model to be estimated simultaneously from all the time series. The advantages of MVAR modeling of multichannel EEG signals in order to compute efficient connectivity estimates have recently been stressed [17-19].

### MultiVariate AutoRegressive models

The approach based on multivariate autoregressive models (MVAR) can simultaneously model a whole set of signals. Let *X* be a set of estimated cortical time series:

 (2)

where *t* refers to time and *N* is the number of cortical areas considered. Given an MVAR process which is an adequate description of the data set *X*:

 (3)

where *X(t)* is the data vector in time; *E(t)=[e_1_(t), …, e_N_]* is a vector of multivariate zero-mean uncorrelated white noise processes; *Λ(1), Λ(2), … Λ(p)* are the *NxN* matrices of model coefficients (*Λ(0)=I);* and *p* is the model order. The *p* order is chosen by means of the Akaike Information Criteria (AIC) for MVAR processes. In order to investigate the spectral properties of the examined process, the Eq. (3) is transformed into the frequency domain:

 (4)

where:

 (5)

and *Δt* is the temporal interval between two samples. Eq. (4) can then be rewritten as:

 (6)

*H(f)* is the transfer matrix of the system, whose element *H_ij_* represents the connection between the* j-th* input and the *i-th* output of the system.

Directed Transfer Function

The Directed Transfer Function, representing the causal influence of the cortical waveform estimated in the *j-th* ROI on that estimated in the *i-th* ROI is defined in terms of elements of the transfer matrix *H*, is:

 (7)

In order to compare the results obtained for cortical waveforms with different power spectra, normalization can be performed by dividing each estimated DTF by the squared sums of all elements of the relevant row, thus obtaining the so-called normalized DTF:

 (8)

where N indicates the number of ROIs, γ^2^_ij_(f) expresses the ratio of influence of the cortical waveform estimated in the* j-th* ROI on the cortical waveform estimated in the *i-th* ROI, with respect to the influence of all the estimated cortical waveforms. Normalized DTF values are in the interval [0 1], and the normalization condition:

 (9)

is applied.

**Figure 2:**
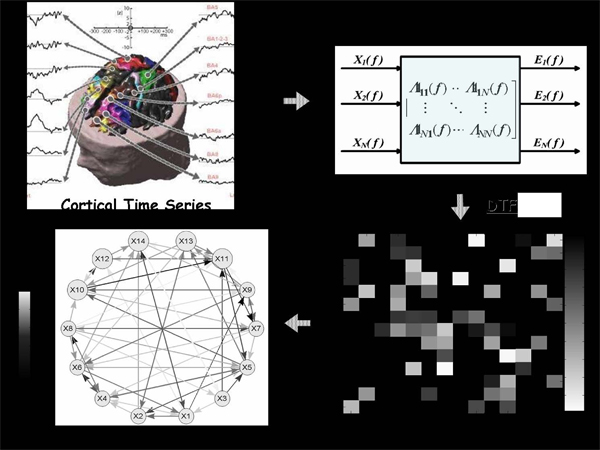
From a set of cortical time series the MVAR method estimates in the frequency domain a functional connectivity pattern that can be modeled by means of a graph.

Figure 2 shows a schematic representation of the functional connectivity estimation from a set of high-resolution EEG signals to the cortical network.

### Graph theory

A graph is an abstract representation of a network. It consists of a set of vertices (or nodes) and a set of edges (or connections) indicating the presence of some of interaction between the vertices. The adjacency matrix *W* contains the information about the connectivity structure of the graph. When a weighted and directed edge exists from the node *i* to *j*, the corresponding entry of the adjacency matrix is *W_ij_* ≠ 0; otherwise *W_ij_* = 0.

### Node strength

The simplest attribute of a node is its connectivity degree, which is the total number of connections with other vertices. In a weighted graph, the natural generalization of the degree of a node *i* is the node strength or node weight or weighted-degree. This quantity has to be split into in-strength *s_in_* and out-strength *s_out_*, when directed relationships are being considered. The strength index integrates the information of the links’ number (degrees) with the connections’ weight, thus representing the total amount of outgoing intensity from a node or incident intensity into it. The formulation of the in-strength index *s_in_* can be introduced as follows:

 (10)

It represents the whole functional flow incoming to the vertex *i*. *V* is the set of the available nodes and *w_ij_* is the weight of the particular arc from the point *j* to the point *i*. In a similar way, for the out-strength:

 (11)

It represents the whole functional flow outgoing from the vertex *i*.

### Strength distributions

For a weighted graph, the arithmetical average of all the nodes’ strengths *<s>* only gives little information about the distributions of the links intensity within the system. Hence, it is useful to introduce *R(s)* as the fraction of vertices in the graph that have strength equal to *s*. In the same way, *R(s)* is the probability that a vertex chosen uniformly at random has weight *= s*. A plot of *R(s)* for any network can be constructed by making a histogram of the vertices’ strength. This histogram represents the strength distribution of the graph and allows a better understanding of the strength allocation in the system. In particular, when dealing with directed graphs, the strength distribution has to be split in order to consider in a separated way the contribution of the incoming and outgoing flows.

### Network structure

Two measures are frequently used to characterize the local and global structure of unweighted graphs: the average shortest path *L* and the clustering index *C*. The former measures the efficiency of the passage of information among the nodes, the latter indicates the tendency of the network to form highly connected clusters of vertices. Recently, a more general setup has been examined in order to investigate weighted networks. In particular, Latora and Marchiori [20] considered weighted networks and defined the efficiency coefficient *e* of the path between two vertices as the inverse of the shortest distance between the vertices (note that in weighted graphs the shortest path is not necessarily the path with the smallest number of edges). In the case where a path does not exist, the distance is infinite and *e* = 0. The average of all the pair-wise efficiencies *e_ij_* is the global-efficiency *E_g_* of the graph. Thus, global-efficiency can be defined as:

 (12)

where *N* is the number of vertices composing the graph. Since the efficiency* e* also applies to disconnected graphs, the local properties of the graph can be characterized by evaluating for every vertex *i* the efficiency coefficients of ***W**i*, which is the sub-graph composed by the neighbors of the node *i*. The local-efficiency *E_l_* is the average of all the sub-graphs global-efficiencies:

 (13)

Since the node *i* does not belong to the sub-graph ***W**i*, this measure reveals the level of fault-tolerance of the system, showing how the communication is efficient between the first neighbors of *i* when *i* is removed. Global- (*E_g_*) and local-efficiency (*E_l_*) were demonstrated to reflect the same properties of the inverse of the average shortest path *1/L* and the clustering index *C*. In addition, this new definition is attractive since it takes into account the full information contained in the weighted links of the graph and provides an elegant solution to handle disconnected vertices.

### Network dynamics

The dynamical properties are calculated by starting self-avoiding random walks from one input node until a given distance *h*. In our approach, all vertices in the network are chosen as the input, one at each time. Since we consider all possible *h*, no arbitrariness is implied.

**Figure 3:**
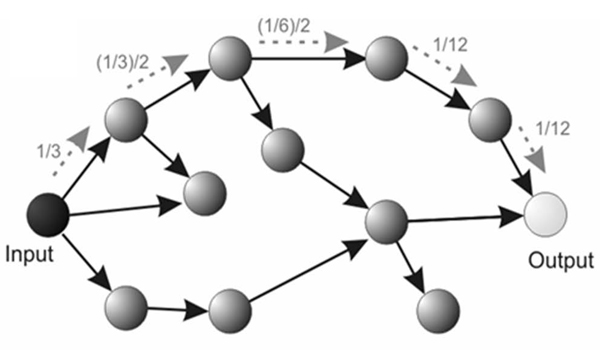
*Up)* Average cortical networks in the Beta band for the SCI group and CTRL group. *Centre)* Location of the ROIs on the realistic cortex model of a representative subject. *Bottom)* The SCI group attempted the foot movement, while the CTRL group executed it.

The network dynamics can be quantified by considering the transition probability *P_h_*(* j*, *i *) between each pair of vertices, which measures the probability of a self-avoiding random walk starting from *i* to reach the vertex *j* after *h* steps. Figure 3 illustrates a possible path of length *h*=5, between the input and output node of a directed graph. Note that such alternative paths are completely overlooked by classical shortest paths length characterization approaches.

The total transition probability of a node *i* over all other nodes by self-avoiding walks of length *h*, is called the *activation*, and it is given by:

 (14)

If a vertex *i* has high activation at distance *h*, it implies that such vertex is connected to a small number of dead-ends distant at once* h* from* i*. Dead-ends are those vertices where the walk cannot propagate, which reduces the activation. The *Ω* index reflects the power of an element to influence other elements in the complex connectivity system. Since it relies on non-preferential random walks, [21] the activation is directly related to diffusion, a type of dynamics that underlies several biological systems including possible spreading of neuronal activation across several cortical regions.

The use of the superedeges approach to investigate the brain connections is motivated because there is a strictly potential relationship between such a methodology and brain organization and function. In the case of dynamics, the activation is related to the brain regions of influence through the network. If a vertex presents a high value of activation for a given distance *h*, it implies that most self-avoiding random walks emerging from such vertex tend to present lengths equal to *h*. Therefore, such vertices tend to have the highest influence along the network, since the signals starting at such vertices tend to propagate through long distances.

The superedges approach implies the consideration of several values of *h* in order to obtain a more complete network characterization. In order to address the problem of having a large number of features to be evaluated (ten subjects and ten different path lengths), we refer to an optimal multivariate statistical method for feature space dimensionality reduction, described as follows.

#### Principal Component Analysis (PCA)

When a large volume of data is available, techniques of dimensionality reduction are necessary. In addition, several modern experiments result in highly redundant databases, which can lead to biases. A possible way to overcome these limitations can be obtained by identifying the principal component analysis methodology, which is a dimensionality reduction transformation that removes data redundancy in an optimal fashion [22] .

Let *X = [x_1_ , x_2_ ,…, x_h_ ] ^T^* be a random vector that represents a set of *h* measured variables. Let *X_i_ , i =1, 2, …, m* , be a sample vector of *m* observations of *X*. In our analysis, *X* would represent each individual and each measured variable, the number of outward paths or outward activation at distance *h*. Given *Z = [ z_1_, z_2_, ... , z_h_]* the *h x h* orthogonal matrix constructed from the eigenvectors of the sample covariance matrix of *X*, then the elements of *z_i_* give the contribution weight of each measurement for the *PCA* component *i*. The new feature vectors can be obtained from the original normalized feature vector by the following transformation [38] :

 (15).

This transformation allows one to project the *m x h* dimensional feature into a new space with reduced dimensionality while yielding completely decorrelated new random variables, which correspond to linear combinations of the original features.

The power of the *PCA* methodology stems from the fact that the principal components of *X*, *{u_i_ : i = 1, 2, ... , h}* are all uncorrelated and that the variance of *u_i_* is given by its eigenvalue *λ_i_*. Because the eigenvalues are arranged in decreasing order, it follows that the component of *X* with largest variance explaining the most of the variation in the data is the component *u_1_* along the *z_1_* direction. Similarly, the next largest variance belongs to the component *u_2_* along *z_2_*.

The investigations about the dynamics of the cortical networks in spinal injured patients and healthy individuals consider the optimal statistical methods (*PCA*) for decorrelation of the heavily correlated measurements and dimensionality reduction. In particular, the number of measurements was the number of experimental subjects i.e. *m=10* (five healthy and five spinal cord injured) and the number of variables was the number of considered path lengths i.e. *h=10*. Eventually, we projected the *m x h* spaces of each frequency band into the main three-dimensional spaces.

#### Network thresholding

Only the connections that were statistically significant (at p<0.001) after a contrast with a surrogate distribution of one thousand DTF values among the same ROIs were considered for the network to be analyzed with graph theory’s tools. The graph indexes were obtained from the threshold networks, maintaining only the information about the presence/absence (i.e. 1/0) of a statistical significant link [9].

### Experimental design

Five healthy (CTRL) subjects and five spinal cord injured (SCI) patients participated to the present study [9]. In particular, spinal cord injuries were of traumatic etiology and located at the cervical level (C6 in three cases, C5 and C7 in two cases, respectively); patients had not suffered for a head or brain lesion associated with the trauma leading to the injury. The informed consent statement was signed by each patient after the explanation of the study, which was approved by the local institutional ethics committee. For the EEG data acquisition, subjects were comfortably seated on a reclining chair, in an electrically shielded and dimly lit room. They were asked to perform a brisk protrusion of their lips while they were performing (healthy subjects) or attempting (SCI patients) a right foot movement. By means of the lips protrusion, the SCI patients provided an evident trigger in correspondence of their attempt to move. For each subject, the cortical activity was estimated according to the high-resolution EEG technique. By using the passage through the Tailairach coordinates system, twelve Regions Of Interest (ROIs) were then obtained by segmentation of the Brodmann areas (B.A.) on the accurate cortical model utilized for each subject. Bilateral ROIs considered in this analysis are the primary motor areas for foot (MIF) and lip movement (MIL), the proper supplementary motor area (SMAp), the standard pre-motor area (BA6), the cingulated motor area (CMA) and the associative area (BA7).

**Figure 4:**
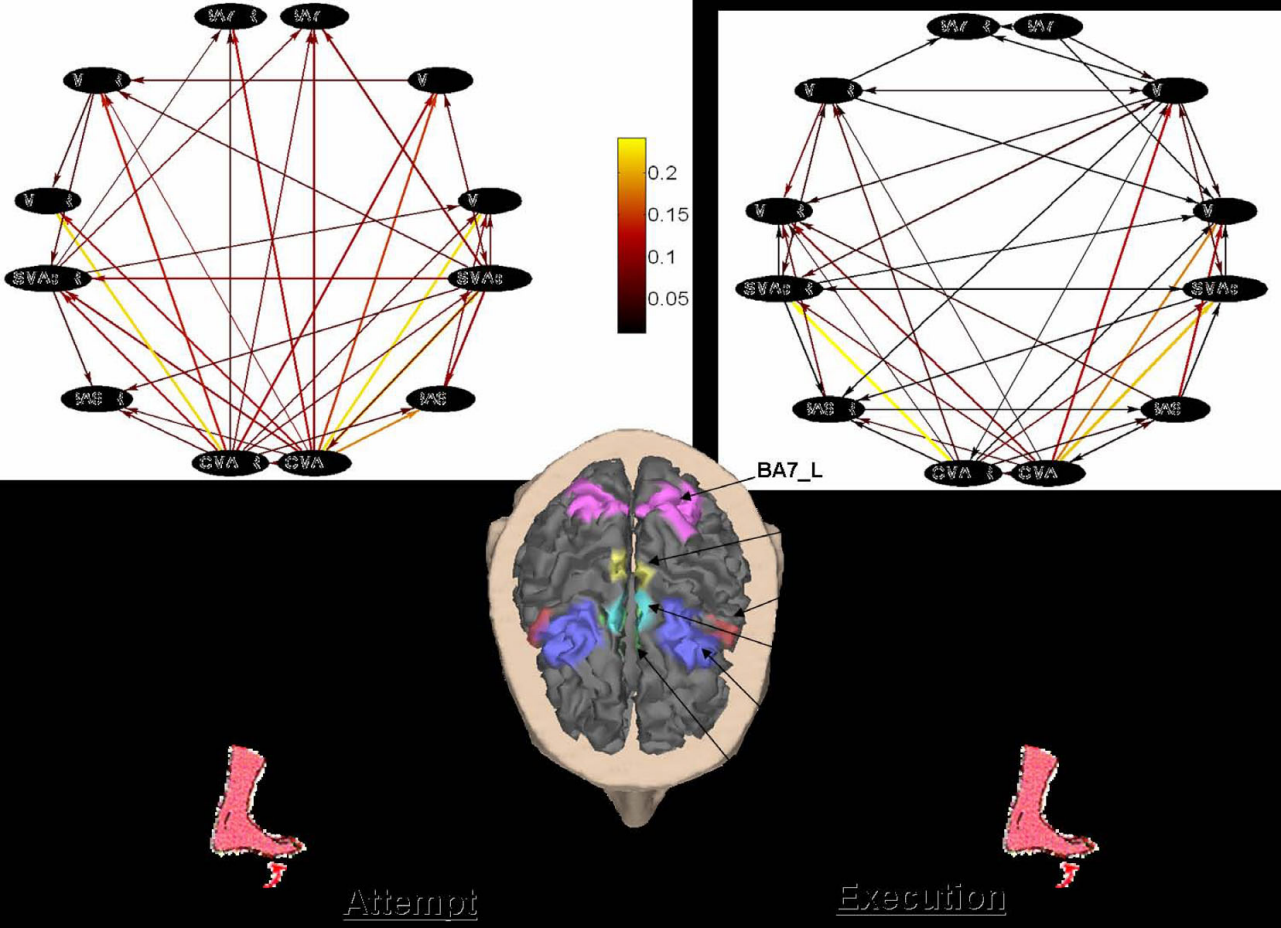
*a)* Average in- and out-degrees for the SCI group in the Beta frequency band.* b)* Average in- and out-degrees for the CTRL group in the Beta frequency band. *c)* Average in- and out-degree distributions for the SCI group in the Beta frequency band.* d)* Average in- and out-degrees distributions for the CTRL group in the Beta frequency band.

In order to study the preparation to an intended foot movement, a time segment of 1.5 seconds before the lips pursing was analyzed. The lips movement was detected by means of an EMG electrode located over the lip muscle. The frequency sampling was 200 Hz for both EEG and EMG signals. EEG signals were referenced to the mean activity from the pre-auricural points A1 and A2. The task was repeated every 6-7 seconds, in a self-paced manner, and the 100 single trials recorded will be used for the estimate of functional connectivity by means of the Directed Transfer Function in four frequency bands (Theta 4-7 Hz, Alpha 8-12 Hz, Beta 13-29 Hz, Gamma 30-40 Hz). Figure 4 shows the original average cortical network estimated in the Beta frequency band for the SCI group and for the CTRL group, during the motor attempt/execution of the task. The twelve ROIs (the nodes of the cortical network) are indicated on the cortex of one representative subject.

## Results

**Figure 5:**
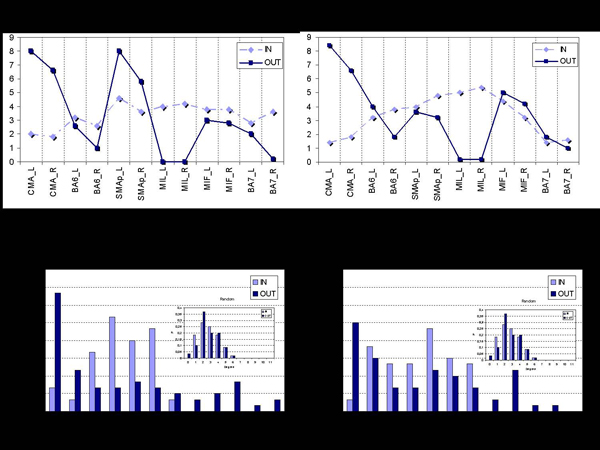
Scatter plot of global- and local-efficiency for SCI networks, CTRL networks and random networks. The Greek symbol codes the average value in a particular frequency band. Black dots identify the values from a distribution of 1000 random graphs.

The upper panels of Figure 5 show the average in- and out-degree in the SCI population a) and in the CTRL group b) for the significant Beta band. Direct comparisons of the data show that in the SCI patients the number of links outgoing from both the SMAp areas Left and Right is largely higher than the CTRL subjects. This result puts in evidence the important role of the supplementary motor areas (SMAp Left and Right) that increase their outgoing functional flows to support the diminished activity of their primary motor areas (MIF Left and Right) during the preparation of this motor act.

The panels at the bottom of Figure 5 show the average profiles of the degree distributions for SCI and CTRL group, in the Beta frequency band. An interesting result is that in-degree and out-degree distributions show different trends within each group.

Right-skew tails of out-degree distributions indicates the presence of few nodes with a very high level of outgoing connections, while for the in-degree distributions there are no ROIs in the network with more than six incoming connections. The inset in each figure illustrates the typical Gaussian profile of the degree-distributions in random graphs, which appears to be different from the estimated cortical networks.

**Figure 6:**
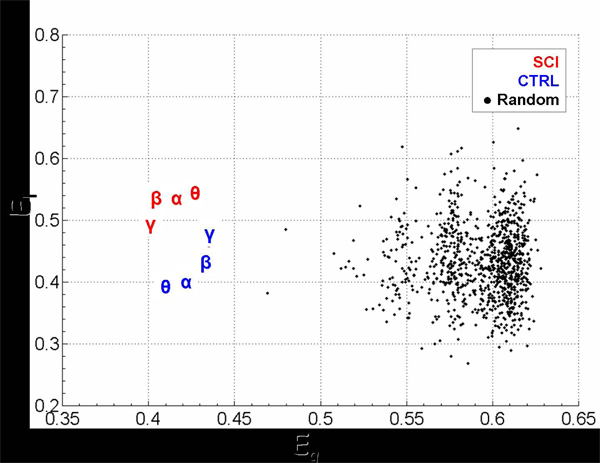
Example of random walk. The arrows indicate possible self-avoiding random walks between the input vertex and the output vertex. The transition probability between the input and output is 1/12. The first probability is calculated by one divided by the number of connections of the input (1/3). At each subsequent step, the probability transition can be obtained by dividing the current probability by the number of non-visited vertices. In the superedges approach, all the possible self-avoiding random walks are considered between the input and output, yielding the respective transition probability.

Fig. 6 shows the contrast between the values of global and local efficiency obtained in the two studied populations with those obtained in a set of one thousand random graphs, having the same number of nodes and arcs.

Analysis of variance (ANOVA p=0.05) was used in order to find significant differences between the indices of efficiency indexes computed in the two groups (SCI, CTRL) for all the frequency bands (Theta, Alpha, Beta and Gamma). ANOVA performed on the global-efficiency *E_g_* variable showed no significant differences for the main factors GROUP and BAND. Instead, the ANOVA performed on the *E_l_* variable revealed a strong influence of the between factor GROUP (F=32.67, p=0.00045); while the BAND factor and the interaction between GROUP X BAND were found not significant (F=0.21 and F=0.91 respectively, p values equal to 0.891 and 0.457). Post-hoc tests revealed a significant difference between the two examined experimental groups (SCI, CTRL) in Theta, Alpha and Beta band (p=0.006, 0.01, 0.03 respectively). It can be observed (Fig. 6) that the average values of the local efficiency in the SCI subjects are significantly higher than those obtained in the CTRL group, for these three frequency bands. Moreover, the estimated cortical networks are not structured like random networks. The statistical contrasts performed by separate Z-tests (Bonferroni corrected for multiple comparisons, p=0.05) were summarized in the Table 1. By inspecting the data presented in both Tab I and Figure 6, it is clear that in general the cortical networks exhibited ordered and regular properties. In particular, the global efficiency is significantly lower than the random mean value, while the local efficiency of the SCI group is significantly higher than random graphs in each band.

**Table 1:** Z-scores of E_g_ and E_l_ from the contrasts with 1000 random graphs.

Z Values	SCI-Theta	SCI-Alpha	SCI-Beta	SCI-Gamma	Healthy-Theta	Healthy-Alpha	Healthy-Beta	Healthy-Gamma
E_g_	-237.45	-250.13	-262.88	-267.07	-249.81	-238.21	-225.95	-223.4
E_l_	57.714	53.314	57.025	38.936	-15.99	-11.051	7.163	21.674

**Figure 7:**
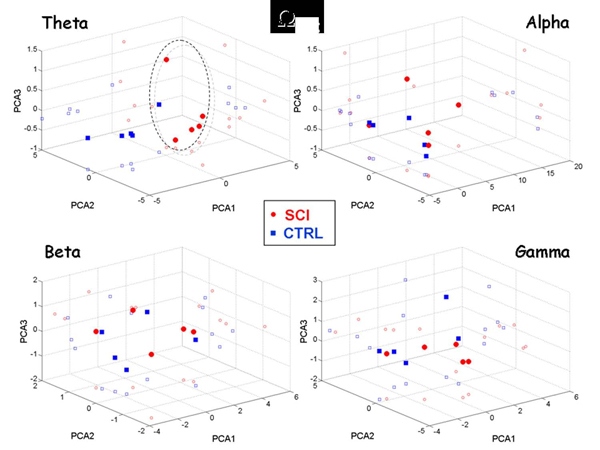
Profile of the activation  in the Theta frequency band. Red circles represent the mean values from the SCI group; blue squares represent the mean values from the CTRL group. Vertical bars indicate the respective standard deviation.

The superedges approach was applied to the estimated cortical networks by considering path lengths ranging from *1* to *10* (*h=1,2…10*, where 10 is the maximum distance observed for such networks). The dynamics of the cortical connectivity patterns was evaluated through the number of outward activations (i.e. the *Ω* index). The results for all the frequency bands are presented in Figure 7. Each scatter plot shows the projections of the *Ω* values with respect to the first three main principal components (i.e. *PCA1, PCA2, PCA3*).

The results show that the separation between *SCI* patients and *CTRL* subjects is clear only for the *Theta* band. The projections obtained for the other bands show several intersections of points between injured patients and healthy subjects. This concludes that the different dynamics generated by self-avoiding random walks in the *SCI* functional network affects mainly the lower spectral contents.

**Figure 8:**
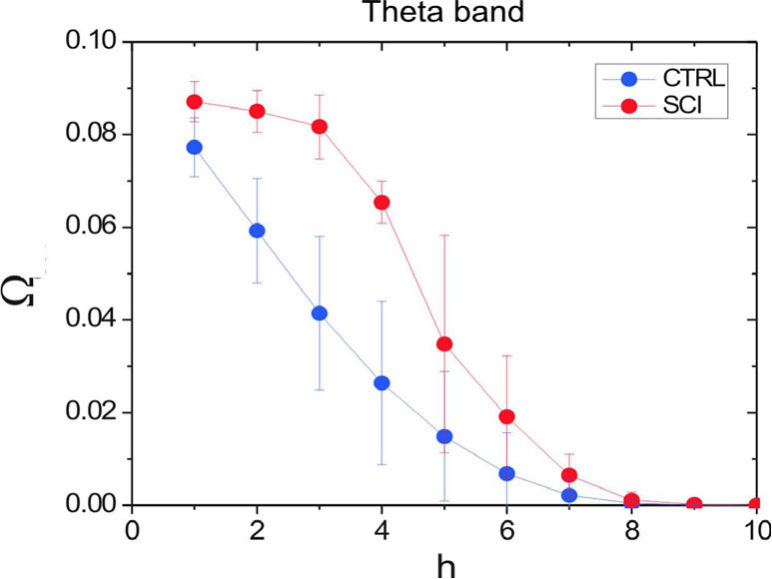
Scatter plot of the three main components obtained through the Principal Components Analysis from the activations of length h=1… 10. Each subplot shows the results found in a different frequency band. Red circles represent values from the SCI group; blue squares stand for values from the CTRL group. Each value is also projected on the respective Cartesian planes (PCA1xPCA2, PCA1xPCA3, PCA2xPCA3).

Figure 8 shows that the mean *Ω* values have similar profiles, with an activation that decreases as *h* increases. This behavior of the activation measurement with respect to *h* indicates that the probability to find dead-ends tends to increase with the distance. In this way, as more distant are the input from the output, fewer random walks departing from the input can reach the output.

However, a clear difference can be observed in the *SCI* network that exhibits a higher activation across the first *h* values (*i.e.1 ≤ h ≤ 5*). We quantified such a difference considering the Manova test. For Rao, Pillai, Lawley-Hotelling and Roy tests, we obtained the *p-*value equal to 0.066, which shows that the *CTRL* and *SCI* networks are different with respect to the activations. Table 2 presents the p*-values* for the other frequency channels.

**Table 2:** Z The p-values for the channels considering Manova (Rao, Pillai, Lawley-Hotelling and Roy tests).

Channel	p-value(*Ω*)
Theta	0.066
Alpha	0.169
Beta	0.705
Gamma	0.632

## Discussion

In the present paper we considered an EEG dataset that has been already studied in previous works [23,24]. The results obtained in those works served as a baseline for the novel approach proposed here. Such approach mainly consists in finding the parallel multiple pathways between cortical areas rather than considering only their shortest paths.

Analysis performed on the cortical networks estimated from the group of normal and SCI patients revealed that both groups present few nodes with a high out-degree value. This property is valid in the networks estimated for all the frequency bands investigated. In particular, cingulate motor areas (CMAs) ROIs act as ‘‘hubs’’ for the outﬂow of information in both groups, SCI and healthy. This means that removal of CMAs from the estimated patterns will cause a collapsing of the whole cortical network, thus corrupting the characteristic behavior of the preparation to the effecting of this experimental task. In addition, while SCI patients show a remarkable ﬂow outgoing from their SMAp areas in the beta frequency band, healthy subjects show a relevant outﬂow from the MIF areas in the same frequency band.

Although the presence of ‘‘hubs’’ in the out-degree distributions of all the cortical digraphs could suggest a power-law trend, we cannot formally assert their scale-free properties, according to actual procedures [25], because the small size of the networks involved prevents us from achieving a reliable degree distributions. Results suggest that spinal cord injuries affect the functional architecture of the cortical network sub-serving the volition of motor acts mainly in its local feature property. In fact, SCI patients have shown signiﬁcant differences from healthy subjects in this index; this could be due to a functional reorganization phenomenon, generally known as brain plasticity [26]. The higher value of local efficiency *E_l_* suggests a larger level of the internal organization and fault tolerance [27]. In particular, this difference can be observed in three frequency bands, theta, alpha and beta, which are already known for their involvement in electrophysiologic phenomena related to the execution of foot movements [28]. A high local efficiency implies that the network tends to form clusters of ROIs which hold an efficient communication. These efficient clusters, noticed in the SCI group, could represent a compensative mechanism as a consequence of the partial alteration in the primary motor areas (MIF) due to the effects of the spinal cord injury. Instead, it seems that the global level of integration between the ROIs within the network do not differ in a significant manner from the healthy behavior. This could mean that spinal cord injuries do not affect the global efficiency of the brain, which attempts to preserve the same external properties observed during the foot-lip task in the cortical networks of healthy subjects. By perusing data presented in both Table 1 and Figure 6, it is clear that cortical networks estimated in this study are also not structured like random networks. Instead, well ordered properties arise from most of the digraphs obtained from each experimental group and frequency band. In fact, they show similar values of global and local efficiency and more precisely fault tolerance is privileged with respect to global communication. Moreover, these real digraphs show a lower global efficiency and a higher local efficiency than respective values obtained from random digraphs. Since the original graphs were rather small (12 nodes), random digraphs are generated by simply shuffling in a random fashion the original links and keeping the same number of nodes. Another way to obtain comparisons that are more robust in large networks can be addressed by using algorithms that also preserve the degree distributions [29].

The *small-world* analysis relies on the estimate of two characteristic measures i.e. the path length and the cluster index. Both these indexes are computed from the shortest paths within the network. The organization of such optimal pathways is very useful as it reveals the level of information processing and signal transmission among different cerebral structures. However, the solely consideration of shortest path distances could provide for an incomplete characterization of networks, since complex connectivity systems with similar shortest paths distribution can indeed exhibit distinct structures and dynamics.

In the present study, we also analyzed the functional network from a different perspective according to a novel approach (i.e. superedges approach) founded on the evaluation of multiple paths between cortical regions. The higher activation observed in the SCI group for the Theta frequency band reflects a lower presence of dead-end *ROIs* that would interrupt the signal propagation toward other cortical areas.

This evidence indicates that the signal propagation within the SCI network is highly increased due to a lower presence of dead-end nodes in the modeled graph. In the literature, *Theta* oscillations have been related to episodic memory process responsible for orientation in space and time [30]. In the light of the results obtained with the standard small-world analysis, a possible interpretation of the increased signal propagation in the *SCI* functional network relies on the need of a higher functional interaction among the ROIs as a mechanism to compensate the lack of feedback from the peripheral nerves to the sensomotor areas.

Eventually, is it worth to note that in the present study the estimation of functional connectivity from EEG measurements is not biased by volume conduction effects. Indeed, it has been proved that the use of the distributed inverse methods (as that used in this paper), as well as the use of cortical imaging, allows to recover the “true” signals at the cortical level from scalp recordings [31,32]. This was proved by using simulations in which it has been tested the degree of accuracy of the reconstruction of the imposed connectivity [33-37]. All these simulations assure that by using a SNR greater than 3 in the data and a more than 15 seconds of data (cumulative on all trials recorded) the reconstruction of the “true” connectivity will be not biased by the volume conduction with errors greater than 5%. These are exactly the conditions in which we employed our methodology from the gathered EEG data.

## Conclusions

The proposed work suggests a possible way to treat the brain connectivity from neuroelectrical measurements with mathematical instruments and methodologies derived from other fields of science. In particular, the present work explored the use of graph theory indexes on the assessment of particular brain functions during movement tasks in tetraplegics. The possibility to estimate such flow of brain networks sub-serving the different functions in the human here explored it is promising for a generation of a better understanding of the brain functions.

## Competing interests

There are no competing interests (financial, political, personal, religious, ideological, academic, intellectual, commercial or any other) to declare in relation to this manuscript.
